# Supplementary figures and images for: Searching for a signature involving 10 genes to predict the survival of patients with acute myelocytic leukemia through a combined multi-omics analysis
Source: PeerJ. 2020 Jun 25;8:e9437. doi: 10.7717/peerj.9437 (PMC7321666; doi:10.7717/peerj.9437)

**A**

CAMK2A

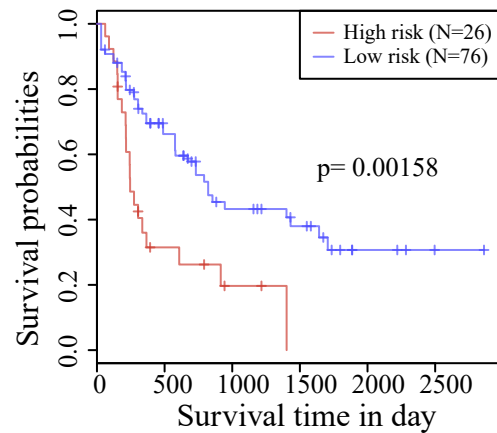**B**

FAT2

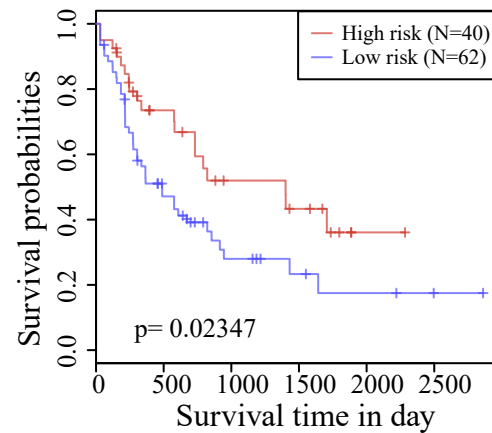**C**

GDF9

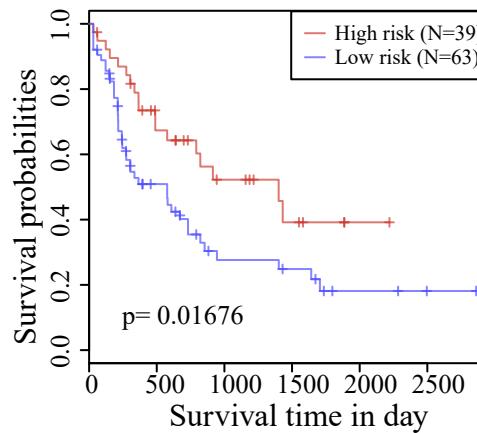**D**

TCERG1

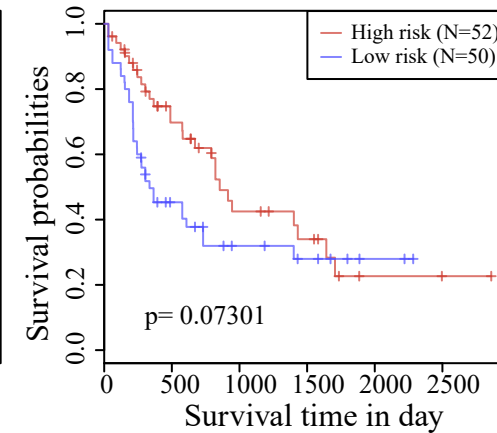**E**

DOC2B

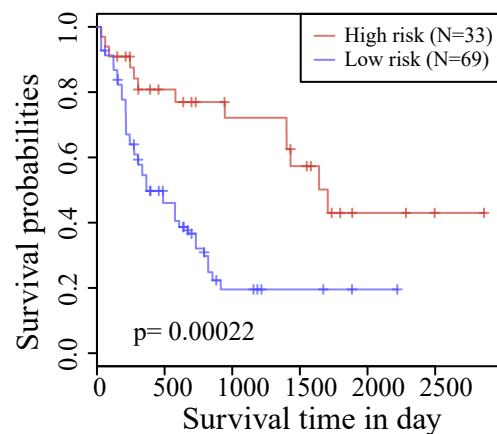**F**

PTGIS

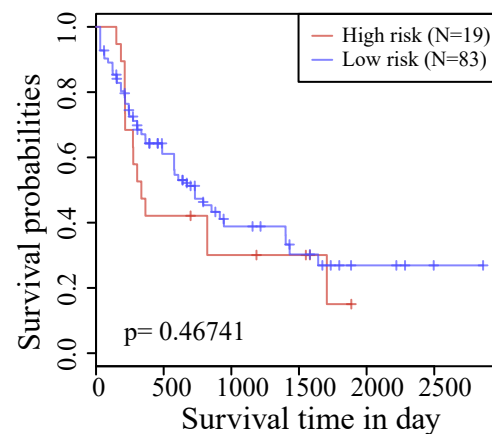**G**

PREX1

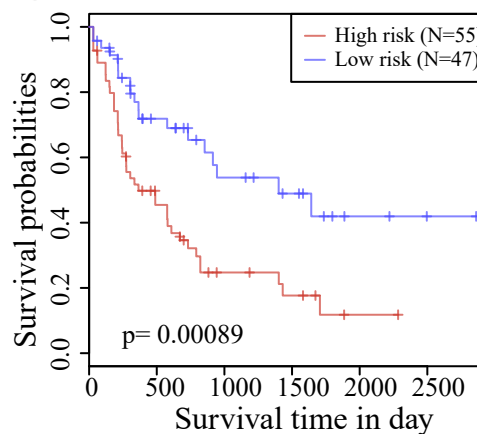**H**

DNTTIP1

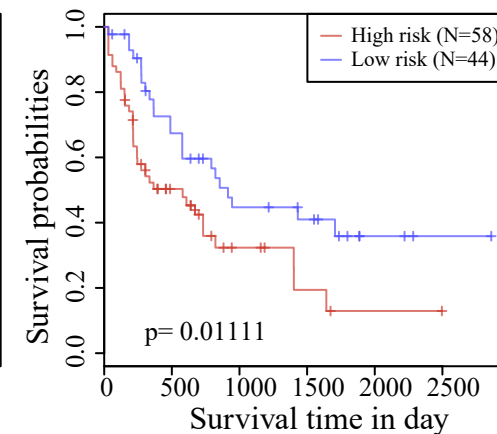**I**

C22orf42

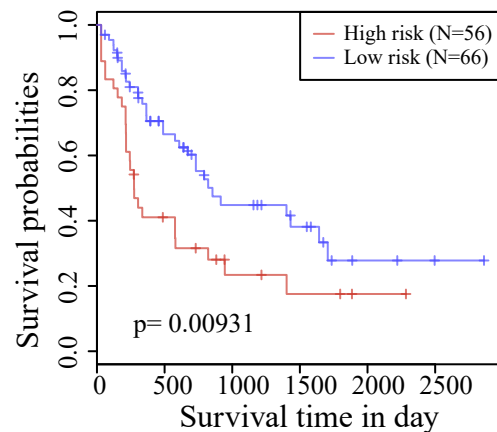**J**

CRISPLD1

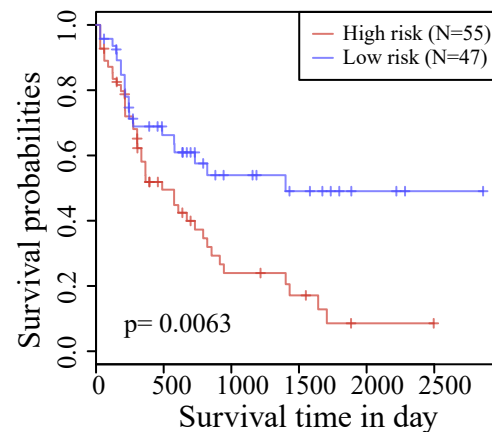

Supplement: Figure S1 [file peerj-08-9437-s001.pdf]

A

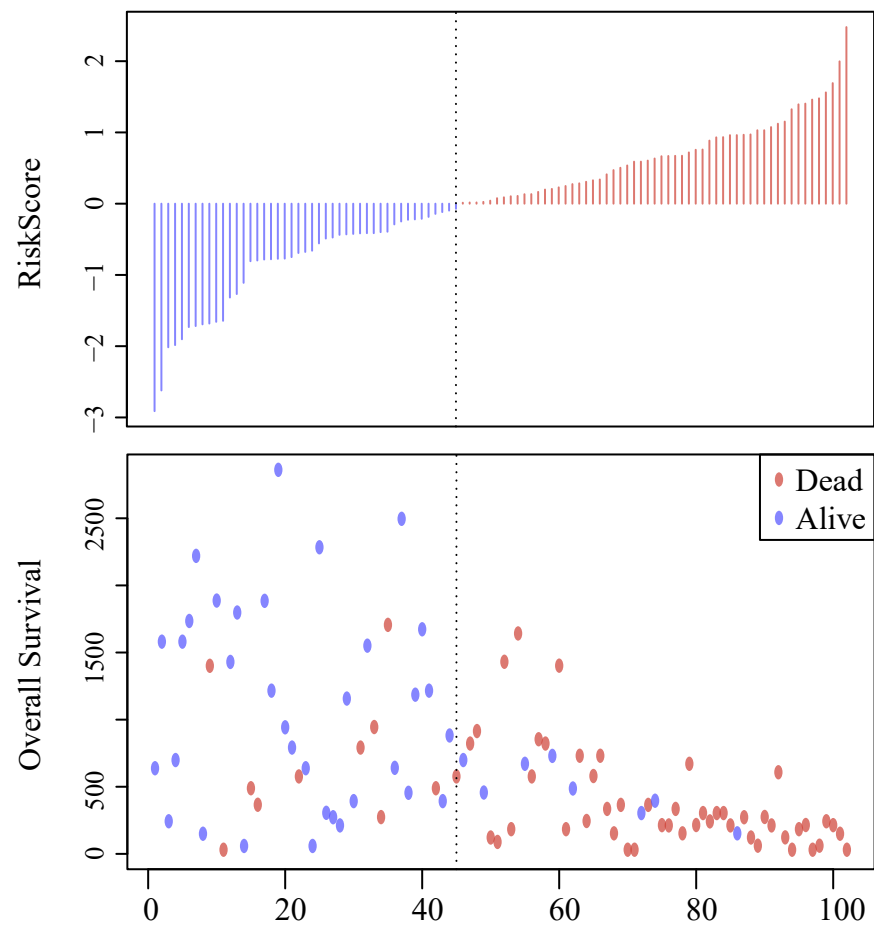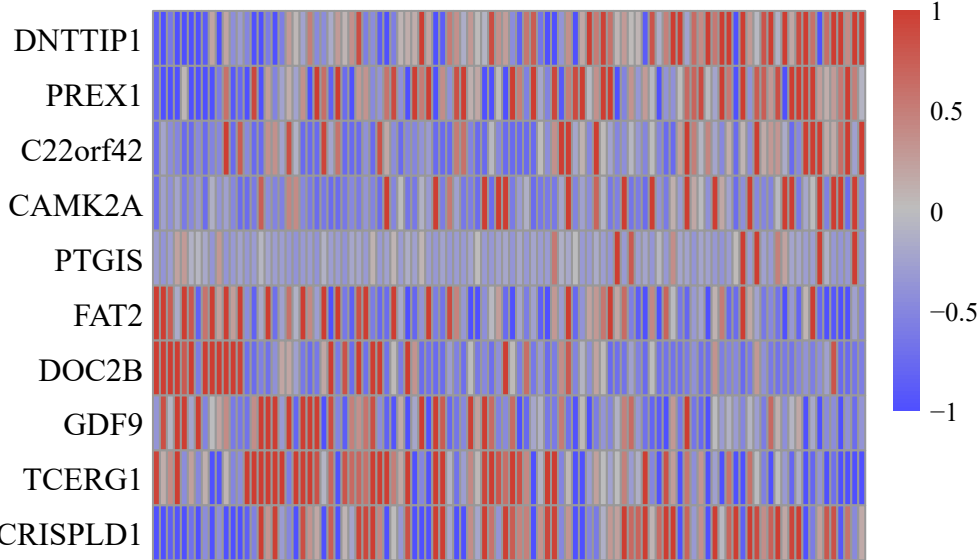

B

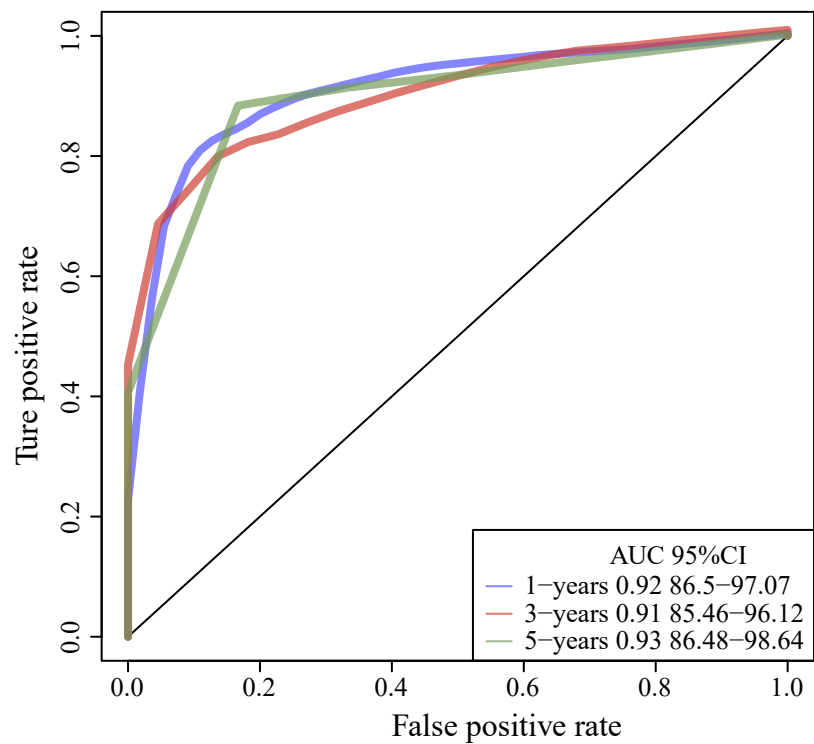

C

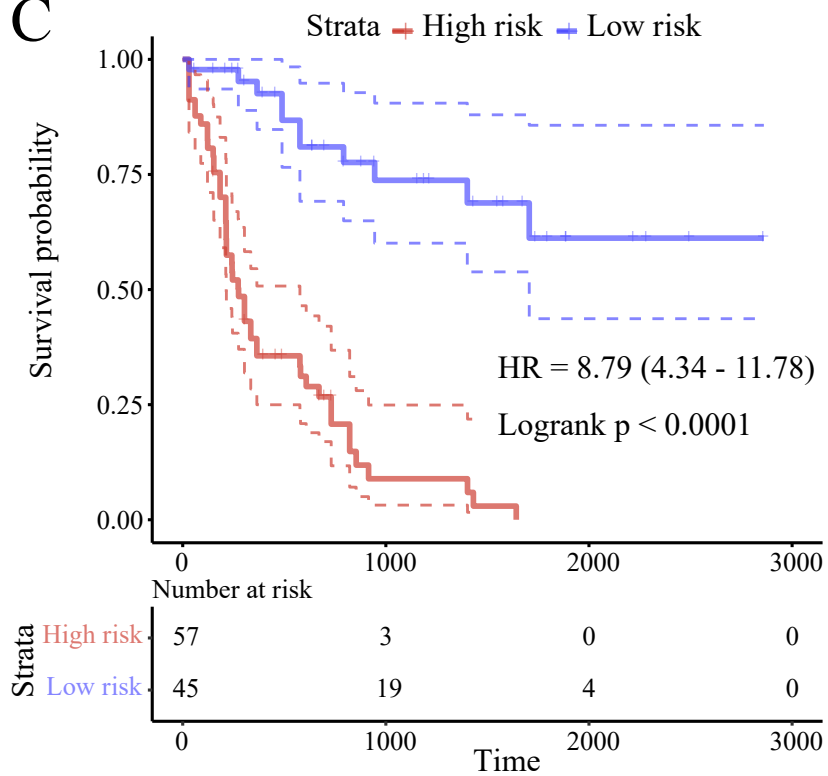

Supplement: Figure S2 — (A) Associations between survival status, survival time as well as risk score and with those 10 genes expression levels within TCGA training set. (B) ROC curves regarding the 10-gene signature for AML samples collected from TCGA training set. (C) Prognostic heterogeneity is analyzed following the estimated classification based on that 10-gene signature within TCGA training set. [file peerj-08-9437-s002.pdf]

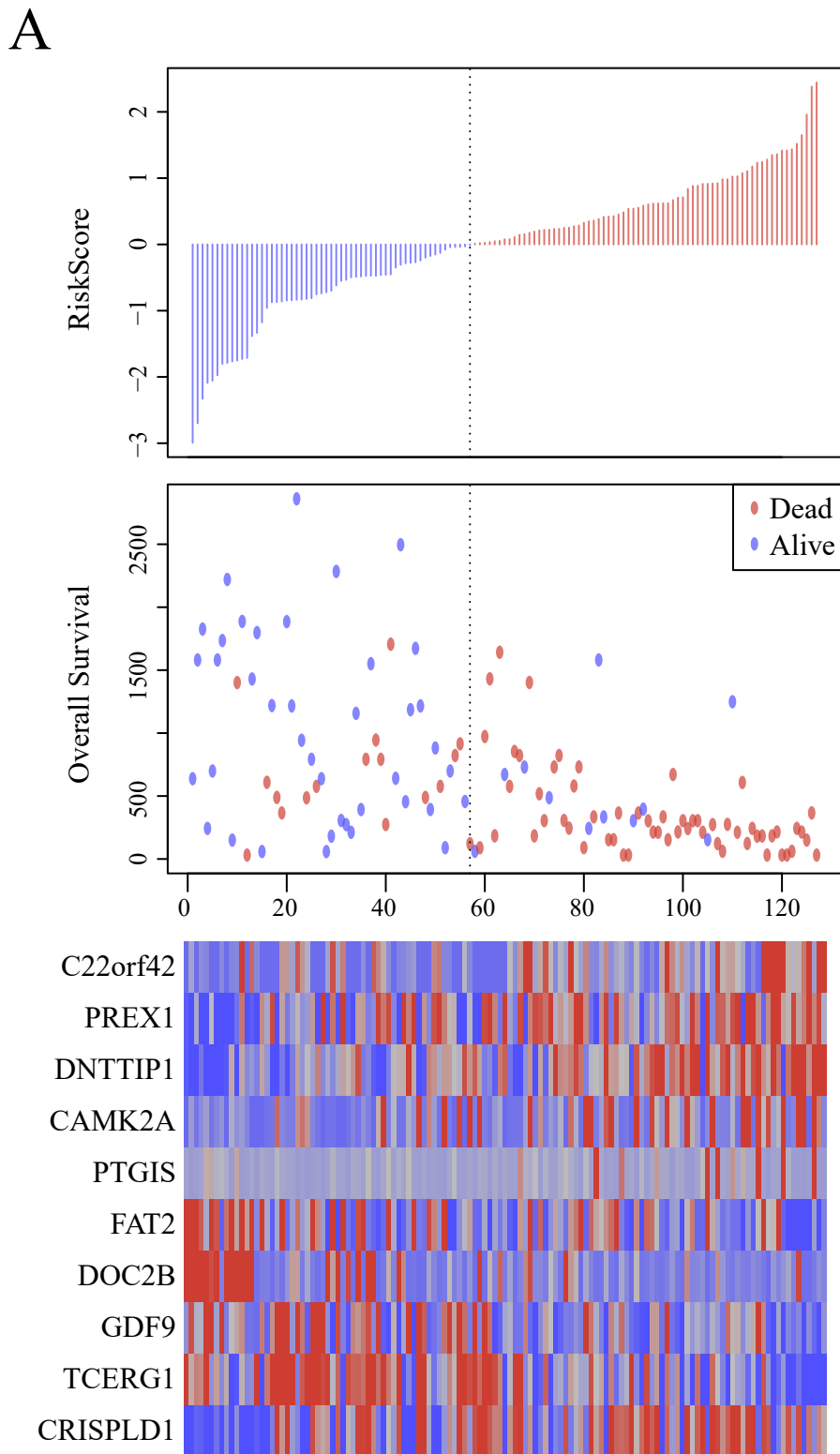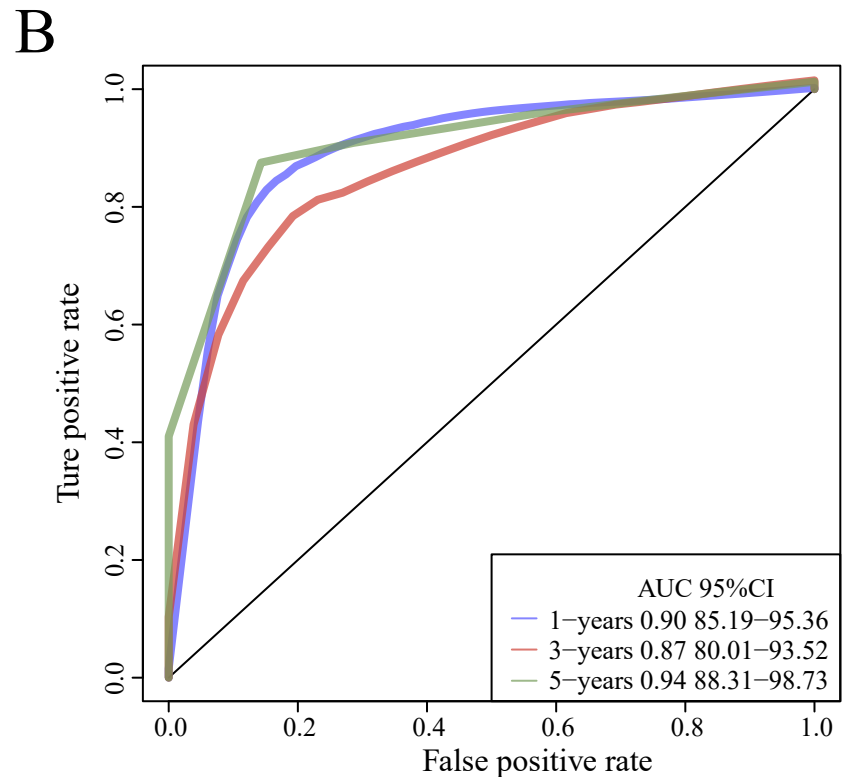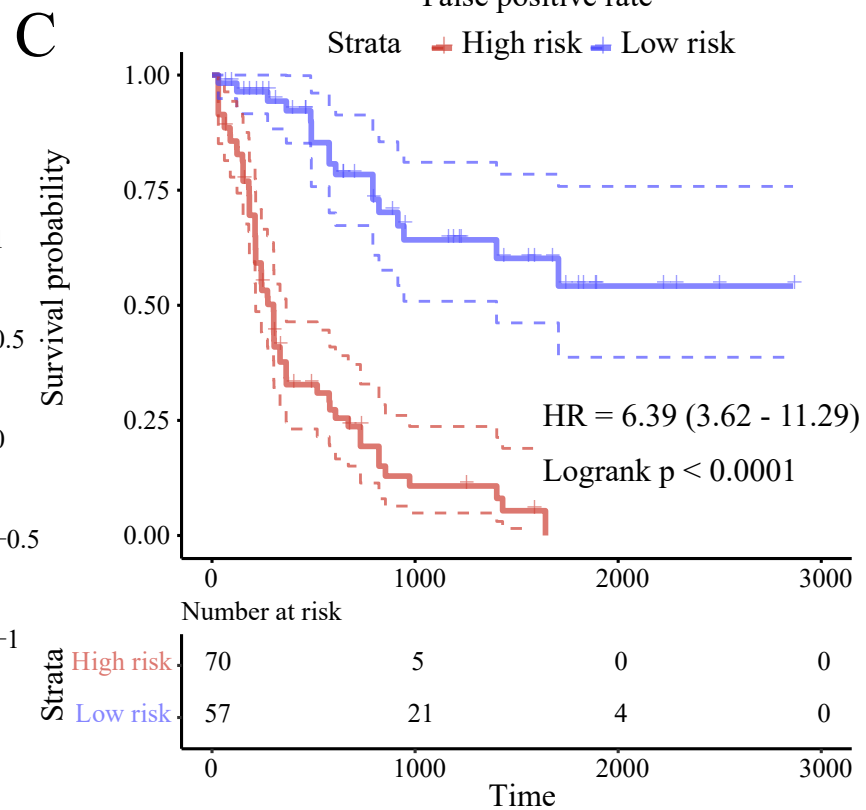

Supplement: Figure S3 — (A) Associations between survival status, survival time as well as risk score and with those 10 genes expression levels within TCGA test set. (B) ROC curves regarding the 10-gene signature for AML samples collected from TCGA test set. (C) Prognostic heterogeneity is analyzed following the estimated classification based on that 10-gene signature within TCGA test set. [file peerj-08-9437-s003.pdf]

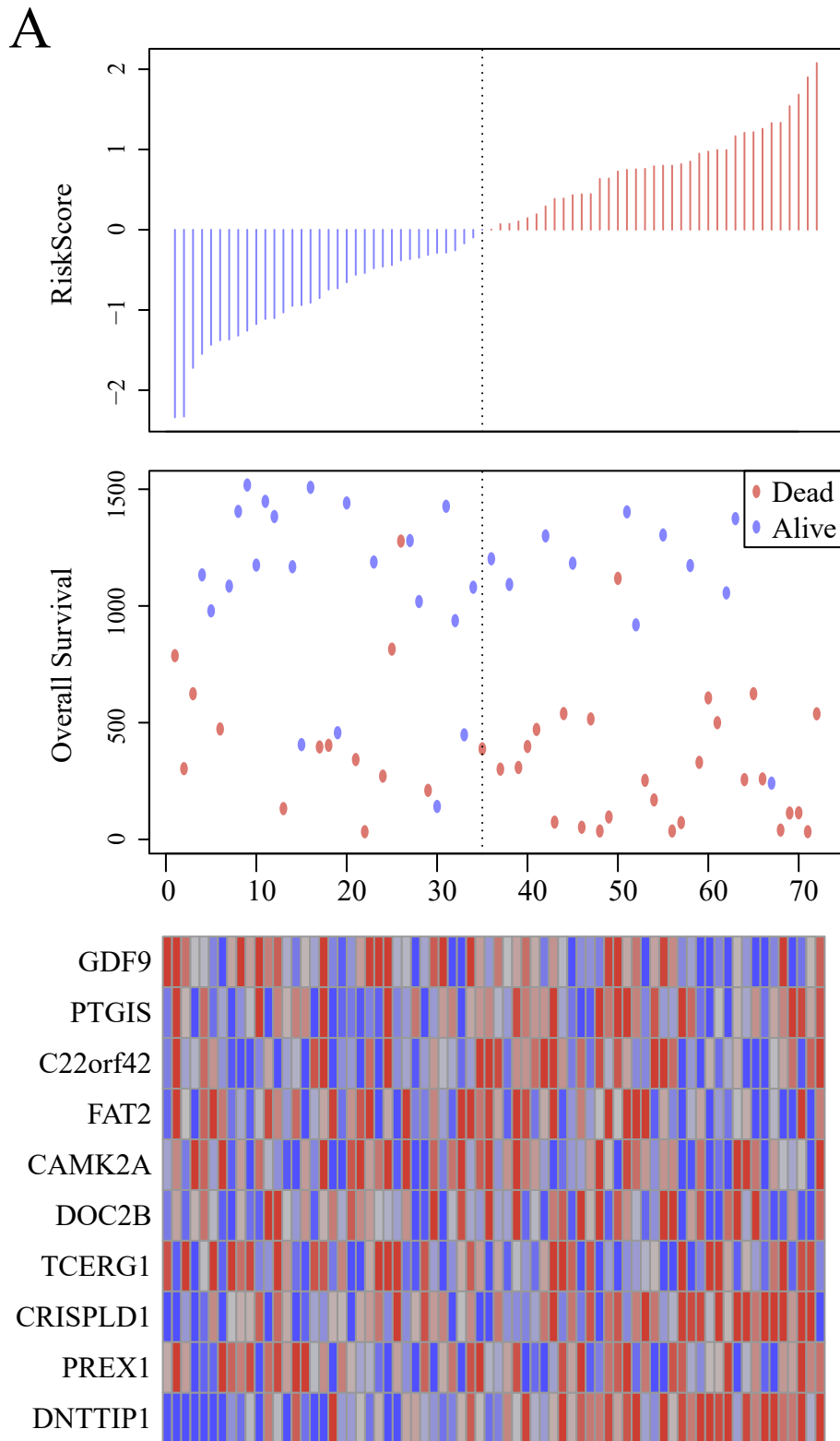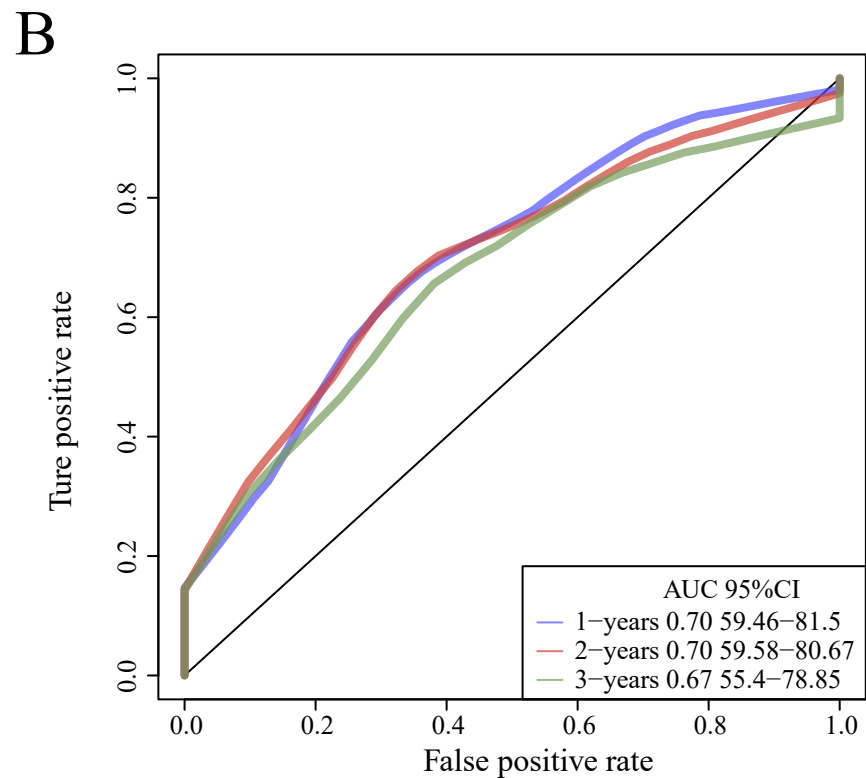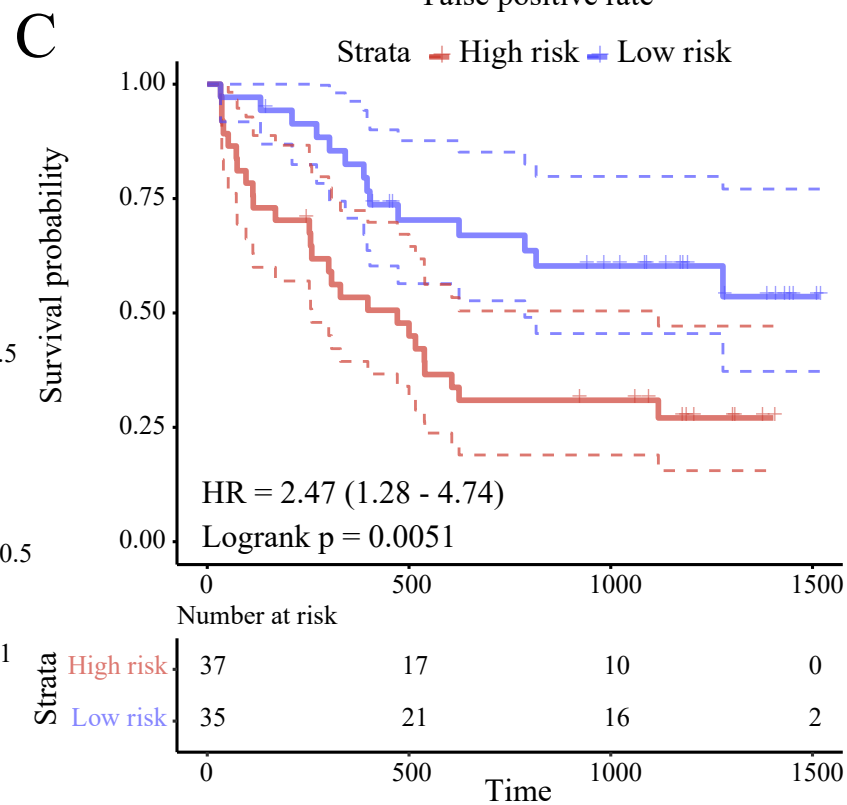

Supplement: Figure S4 — (A) Associations between survival status, survival time as well as risk score and with those 10 genes expression levels within GSE12417 external data set. (B) ROC curves concerning the 10-gene signature for AML samples collected from GSE12417 external data set. (C) Prognostic heterogeneity is analyzed following the estimated classification based on that 10-gene signature with GSE12417 external data set. [file peerj-08-9437-s004.pdf]

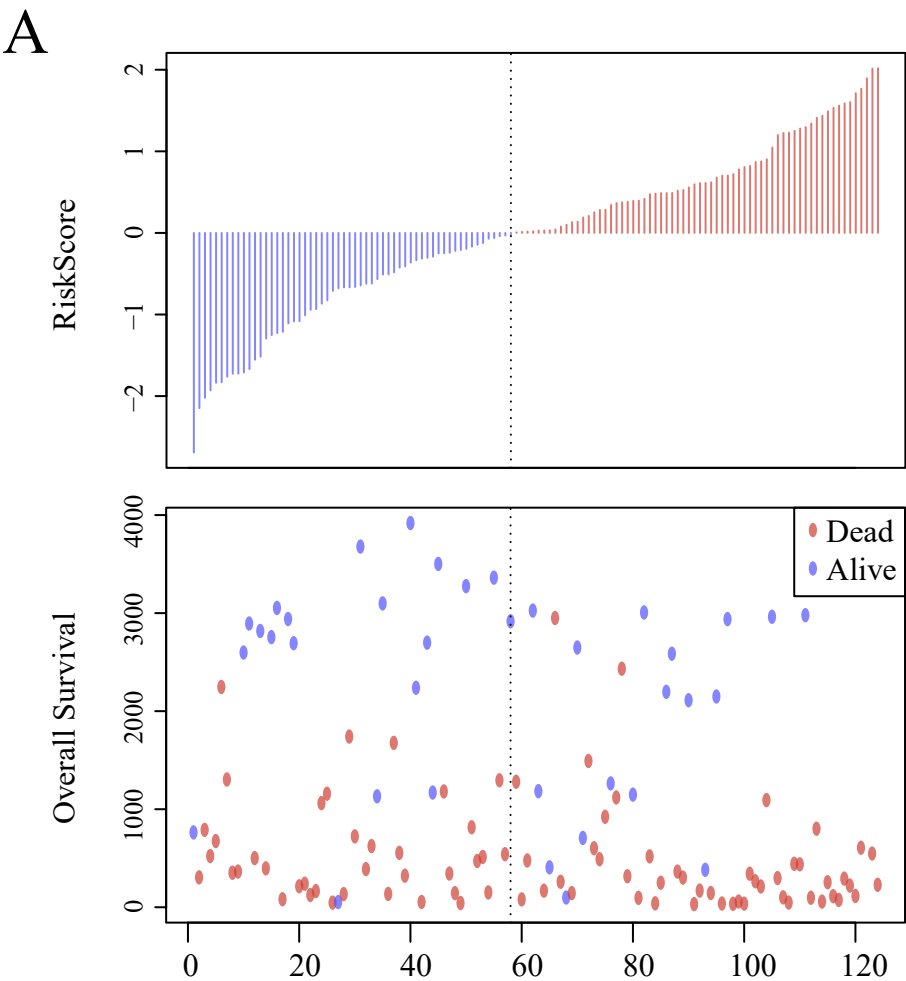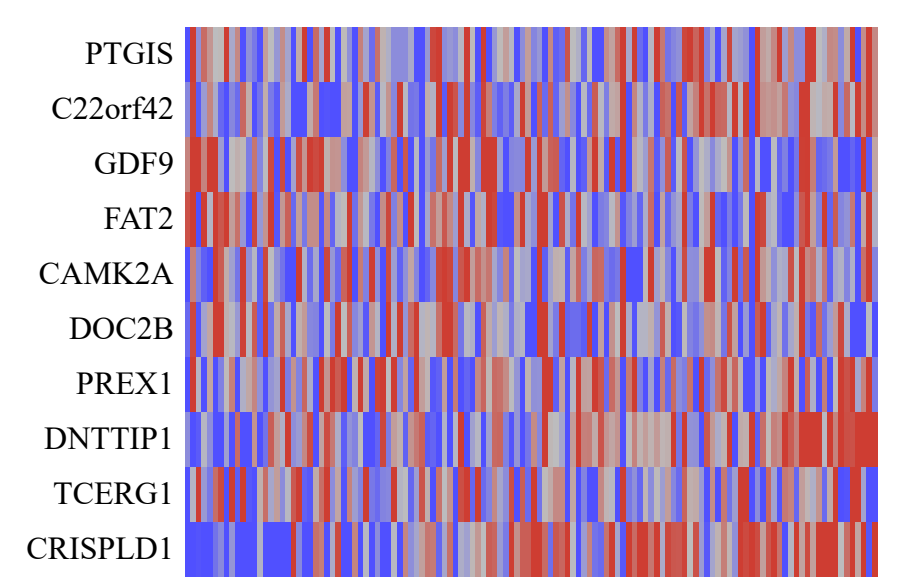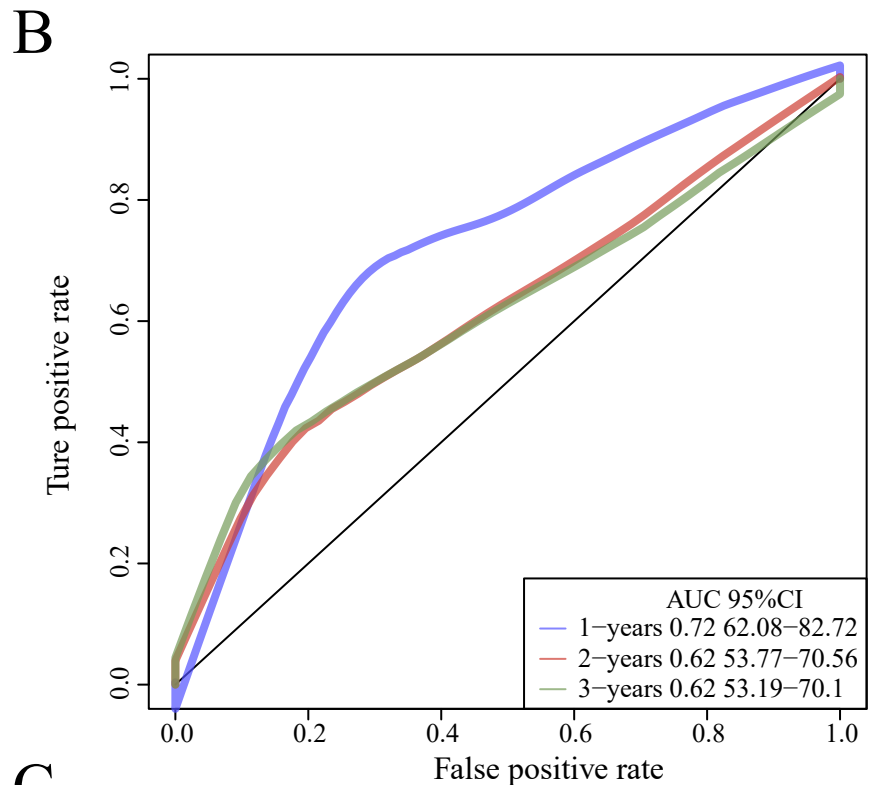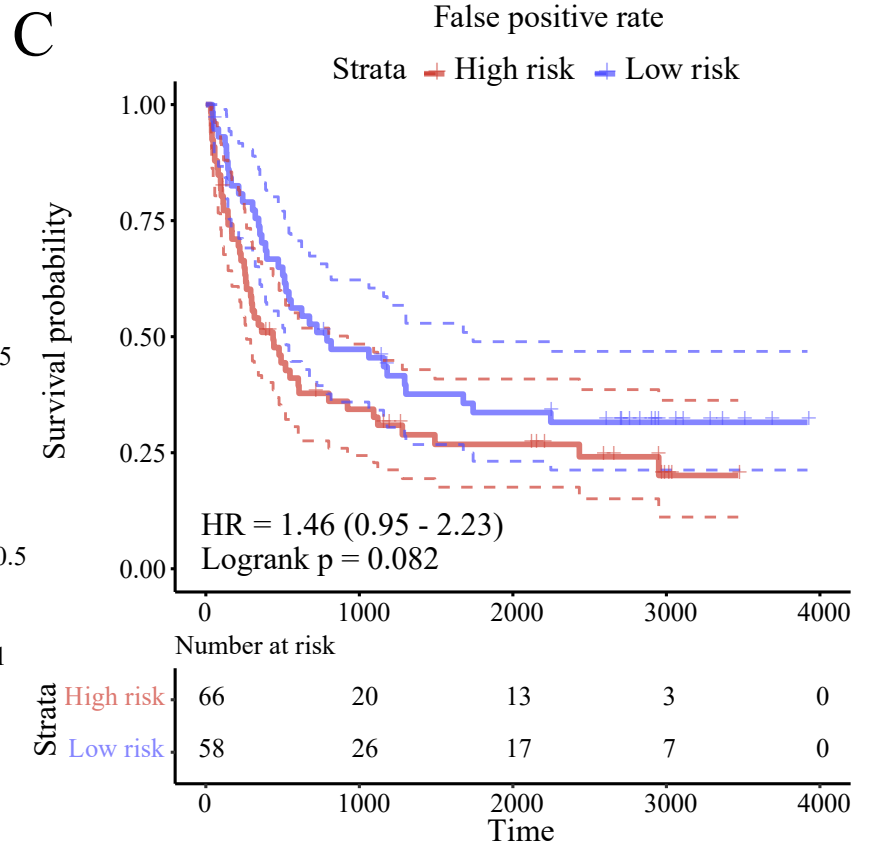

Supplement: Figure S5 — (A) Associations between survival status, survival time as well as risk score and with those 10 genes expression levels within GSE37642 external data set. (B) ROC curves concerning the 10-gene signature for AML samples collected from GSE37642 external data set. (C) Prognostic heterogeneity is analyzed following the estimated classification based on that 10-gene signature with GSE37642 external data set. [file peerj-08-9437-s005.pdf]

**A**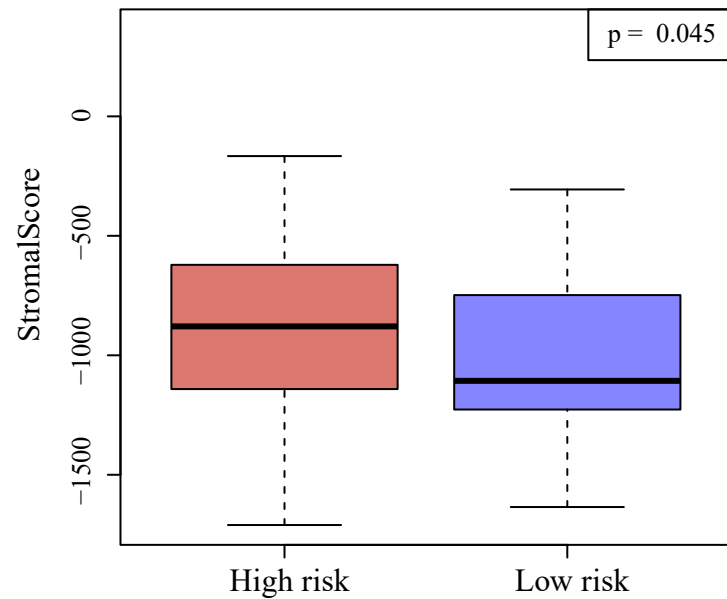**B**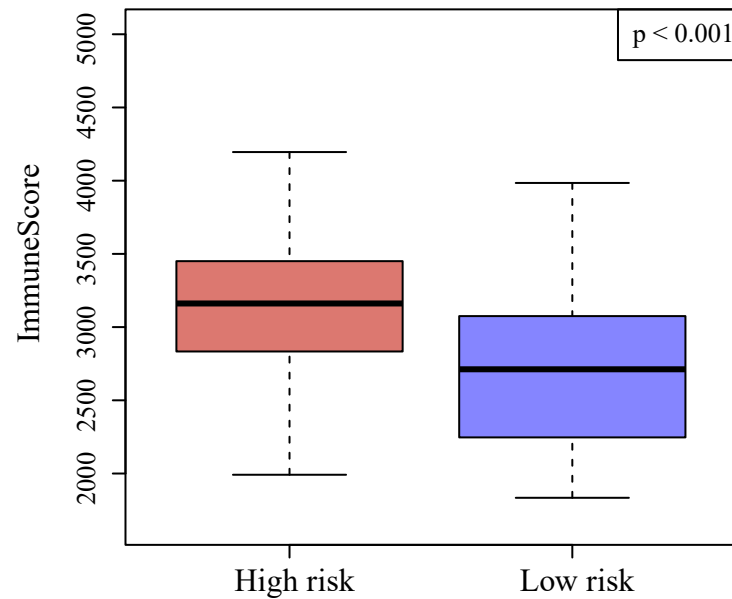**C**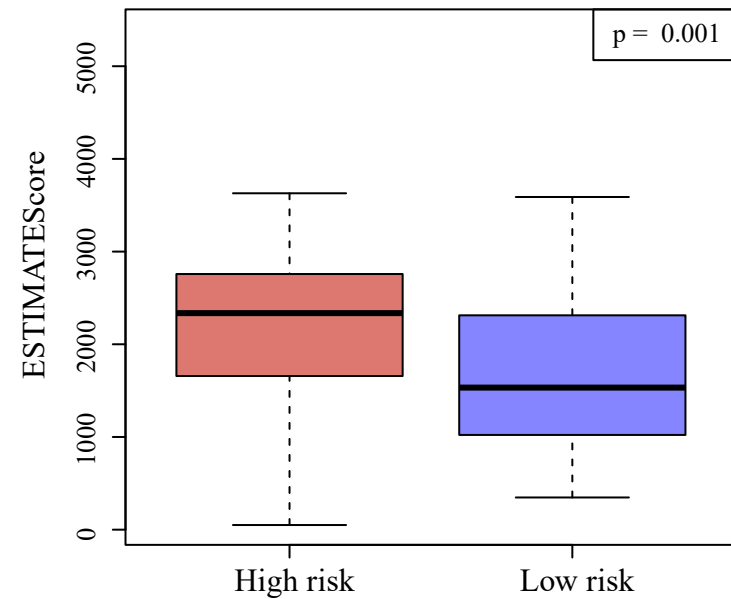

Supplement: Figure S6 — StromalScore (A), ImmuneScore (B) and ESTIMATEScore (C) [file peerj-08-9437-s006.pdf]
